# Recent progress in functional metal–organic frameworks for bio-medical application

**DOI:** 10.1093/rb/rbad115

**Published:** 2023-12-23

**Authors:** Wenwen Chai, Xiaochen Chen, Jing Liu, Liyan Zhang, Chunyu Liu, Li Li, John Robert Honiball, Haobo Pan, Xu Cui, Deping Wang

**Affiliations:** School of Materials Science and Engineering, Tongji University, Shanghai 201804, China; School of Materials Science and Engineering, Tongji University, Shanghai 201804, China; Shenzhen Institute of Advanced Technology, Chinese Academy of Sciences, Shenzhen 518055, China; School of Materials Science and Engineering, Tongji University, Shanghai 201804, China; Shenzhen Institute of Advanced Technology, Chinese Academy of Sciences, Shenzhen 518055, China; Shenzhen Institute of Advanced Technology, Chinese Academy of Sciences, Shenzhen 518055, China; Department of Orthopaedics & Traumatology, School of Clinical Medicine, Li Ka Shing Faculty of Medicine, The University of Hong Kong, Hong Kong, China; Department of Orthopaedics & Traumatology, School of Clinical Medicine, Li Ka Shing Faculty of Medicine, The University of Hong Kong, Hong Kong, China; Shenzhen Institute of Advanced Technology, Chinese Academy of Sciences, Shenzhen 518055, China; Shenzhen Institute of Advanced Technology, Chinese Academy of Sciences, Shenzhen 518055, China; School of Materials Science and Engineering, Tongji University, Shanghai 201804, China

**Keywords:** metal–organic frameworks, porous, functionalization, tissue repair, disease treatment

## Abstract

Metal–organic frameworks (MOFs) have a high specific surface area, adjustable pores and can be used to obtain functional porous materials with diverse and well-ordered structures through coordination and self-assembly, which has intrigued wide interest in a broad range of disciplines. In the arena of biomedical engineering, the functionalized modification of MOFs has produced drug carriers with excellent dispersion and functionalities such as target delivery and response release, with promising applications in bio-detection, disease therapy, tissue healing, and other areas. This review summarizes the present state of research on the functionalization of MOFs by physical binding or chemical cross-linking of small molecules, polymers, biomacromolecules, and hydrogels and evaluates the role and approach of MOFs functionalization in boosting the reactivity of materials. On this basis, research on the application of functionalized MOFs composites in biomedical engineering fields such as drug delivery, tissue repair, disease treatment, bio-detection and imaging is surveyed, and the development trend and application prospects of functionalized MOFs as an important new class of biomedical materials in the biomedical field are anticipated, which may provide some inspiration and reference for further development of MOF for bio-medical applications.

## Graphical abstract

**Figure:**
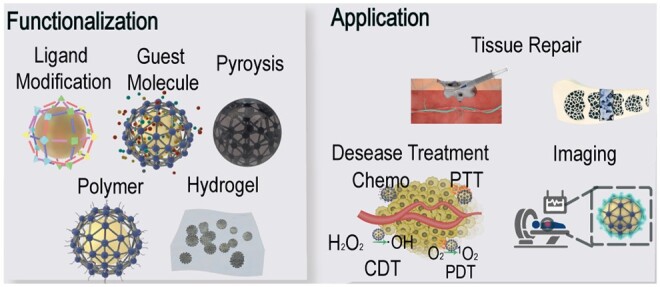

## Introduction

As a class of coordination polymers, metal–organic framework (MOF) materials were originally proposed by Yaghi in the 1990s. This emerging material, with a carefully designed topological structure, has attracted widespread public interest including, but not limited to, applications in energy storage and conversion, photo/electrical catalysis, and bio-medicine [1–4]. As a new organic–inorganic hybrid material, MOFs have garnered a great deal of interest due to their diverse structures, high specific surface area and high porosity and have demonstrated promising applications in a variety of fields including catalysis, gas adsorption/separation/storage, optics, sensing and detection, energy, and biomedicine [5–11]. A typical MOF comprised metallic nodes and organic ligands. The former includes transition and lanthanide metal ions, while the latter contains carboxylates, phosphates, imidazole and pyridine nitrogen-containing heterocyclic ligands, porphyrins, cyclodextrin bioligands, etc. [5, 12–14]. There are several varieties of MOFs including the University in Oslo (UiO) series of carboxylic acid ligands, the Materials of Institute Lavoisier (MIL) frameworks series and the zeolitic imidazolate frameworks (ZIF) series of nitrogen-containing heterocyclic ligands, among others [13, 15–17]. Numerous techniques, including hydrothermal/solventothermal synthesis, kettle thermal methods, microwave methods, microemulsion methods, ball milling and template methods, can all be employed to generate a vast array of MOFs and their composites [3, 6]. Thousands of MOFs with variable morphologies, compositions, sizes, and physical and chemical characteristics are created by combining different metal ions/clusters with organic ligands [4]. MOF pores may be engineered with varying distributions or states to fulfill the specific requirements for several applications.

Due to the compositional flexibility of MOF materials, MOFs may be specifically tailored for their desired application. There exists a vast selection of ligands and nodes, allowing various combinations to be produced by MOFs with distinct functionalities [18–22]. In addition, ligand units and nodes may supply additional reactive sites, allowing for the loading of functional molecules [23–25]. Furthermore, MOFs may be incorporated into hydrogels through physical or chemical interactions, where hydrogels characteristically contain a high-water content, outstanding flexibility and good biocompatibility, holding significant potential for use in biomedical-related applications [26–28].

**Figure 1.**
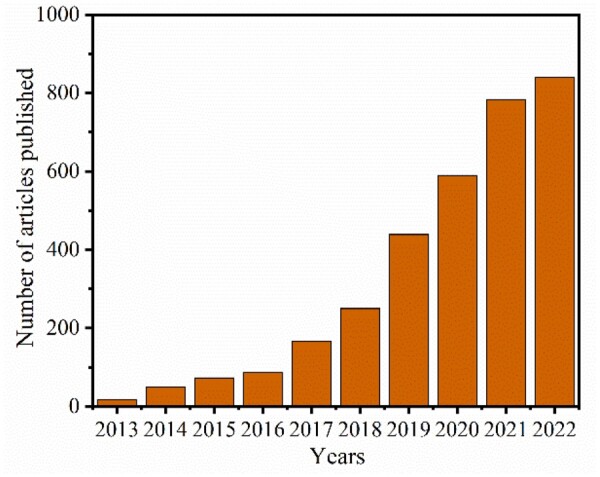
Statistics of publications with the topics including “metal organic framework and tissue regeneration, disease therapy, biological detection or biological imaging” in the web of science search conducted on May 16, 2023.

In terms of biomedical engineering, functional MOFs are widely used in a variety of contexts including drug delivery, enzyme or cell activity protection, antibacterial, bioimaging, biocatalysis and biosensing [29–37]. Recent trends in the application of MOF materials in the biomedical field indicate its promising research in this domain (Figure 1). Functional MOF materials provide ideal drug cages due to their high specific surface area and porosity, as well as their biocompatibility and chemical applicability. Importantly, certain MOF compounds can respond to external physical or chemical stimuli, allowing the release of medicinal molecules to facilitate several tissue healing applications on-demand. In addition, functionalized MOF frames can inhibit or even remove tumors by utilizing phototherapy, chemodynamic therapy, starvation therapy, immunotherapy, drug therapy and sonodynamic therapy [29–35]. On this basis, functionalized MOFs can serve as a potential material for multi-modality combination therapy for efficient tumor clearance [36–38]. Moreover, functionalized MOFs may be applied for tumor clearance in addition to *in vivo* imaging to better clarify the localization and size of tumors, which is essential for achieving accurate clinical diagnostic treatment integration strategies [39–45]. MOFs represent a vital bridge between the disciplines of bioengineering and material science, playing a significant role in the advancement of biomaterial treatment integration. However, there exists only a few review articles which primarily focus on the application of MOFs for biomedical applications.

In this work, several functionalization procedures including multi-metal node/ligand design, pyrolysis, guest molecular, polymer grafting and hydrogel integration have been examined in detail. Based on this, we have placed focus on the present state of research on the functionalized modification of MOFs and their application in the field of biomedical engineering, which is examined in connection to the technological advancement trends in related areas.

## Multi-functionalization of MOFs

One of the most intriguing characteristics of MOFs is that functionalized modification enhances the utility of MOF materials without compromising their inherent features [46–50].

### Principle of MOF coordination

For functionalization of diverse MOFs, the stability of MOFs should be prioritized, since it affects mostly on modification process and the subsequent outcomes. And the binding ability between organic ligands and metal nodes is one of the most predominant factors, which can be explicated by front-line orbital based hard–soft acid–base (HSAB) theory [51, 52]. HSAB categorizes acids and bases into: hard, soft and in-between neutral acids and bases. To be specific, hard acids are metal ions with small ionic radii, high oxidation states and low polarizability, such as Cr^3+^ and Zr^4+^. Meanwhile, hard bases are organic ligands with high electronegativity, low polarizability, and are not easy to oxidize, such as various carboxylic acid ligands. For example, in the case of soft acid–soft base coordination process, the valence electrons are shared by the highest occupied orbital (HOMO) and the lowest empty orbital (LUMO) and this bound is dominated by covalent bonding properties. Otherwise, in the case of the hard acid–hard base, the coordination has ionic bonding properties. Common hard acids include Zr^4+^, Fe^3+^, Mg^2+^, Ca^2+^, neutral acids such as Cu^2+^, Fe^2+^, Sn^2+^, Mn^2+^, and soft acids such as Cu^+^, Ag^+^. Hard base organic ligands include homophthalic acid, terephthalic acid, neutral bases such as pyridine, aniline, soft bases such as imidazole, adenine [52, 53].

Generally, hard acid–base and soft acid–base presented better stability than that of other combinations [54]. The metal ions and ligands exchange strategy is intended to enhance the stability of the MOFs [55].

Functionalized modification approaches for MOFs include guest molecules, polymers, biomolecules (peptides, proteins, nucleic acids, polysaccharides), hydrogels and carbonization [56–62]. Each of these strategies will be detailed in the parts that follow.

### Bimetallic/biligand MOFs

Additional metal ions or organic groups may coexist with the initial MOF structure in order to modulate porosity, hydrophobicity, active sites, etc. [63–65]. These structures are often referred to as bimetallic, biligand and bimetallic/biligand MOFs. Extra coordination metals with similar physicochemical properties to the host metal in the parent MOFs give more stable coordination structures. The transition group metals, such as Mn, Fe, Co, Ni, Cu and Zn, have traditionally been the most widely investigated metals for bimetallic/biligand MOF modification [66–72]. Meanwhile, depending on the size difference between the main and secondary ligand, the supplementary ligand may either enter the framework or replace the parent outermost ligand [73–75].

The bimetallic coordination MOFs may be created in a single step by the reaction of two metal ions with the ligand solution. Yu *et al*. [76] developed a bimetallic MOF comprising Zn and Gd ions in a single hydrothermal procedure. According to structural analysis, the micromorphology was successfully preserved after Gd doping and the Zn ions were partially replaced by Gd^3+^, consequently endowing the Zn-MOF with an increased immunotherapy effect for the treatment of cancer.

**Figure 2.**
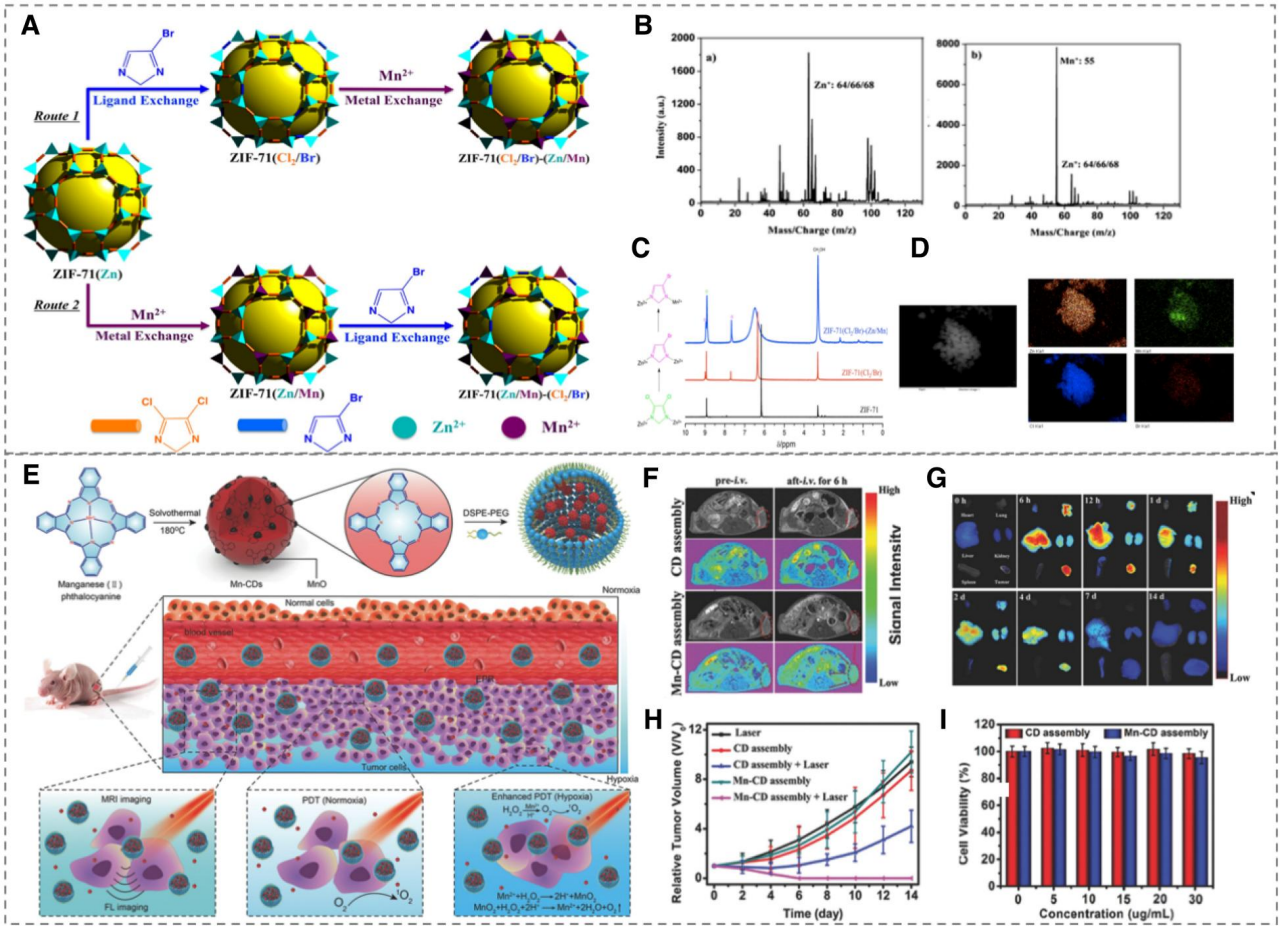
**(A)** Schematic diagram of the stepwise PSE modification of ZIF-71 with Mn^2+^ and 4-Bromo-1H-imidazole, **(B)** positive ion spectra of ATOFMS and **(C)** 1H NMR before and after exchange, **(D)** SEM-EDX mapping of ZIF-71(Zn/Mn)-(Cl_2_/Br)]. Reproduced from Ref. [73] with permission of American Chemical Society, ©2013. **(E)** schematic of the Mn-CD preparation and its use to improve tumor hypoxia and increase PDT efficiency, **(F)** MR images of nude mice and **(G)** FL images of major organs after injection of Mn-CD, **(H)** tumor growth curves of nude mice after different treatments, **(I)** relative viability of Mn-CD cultured HeLa cells. Reproduced from Ref. [95] with permission of John Wiley and Sons, ©2018.

Post-synthetic exchange (PSE) is also often used to produce binary metal/ligand MOFs [64, 77–80]. As depicted in Figure 2(A), Cohen *et al*. [73] grafted Mn^2+^ and 4-Bromo-1H-imidazole ligands into ZIF-71 particles using the PSE technique with negligible effects on the original particle size, morphology, thermal stability and specific surface area, indicating great potential for regulating the specific properties of MOFs. Furthermore, Du *et al*. [74] employed a phthalic acid ligand to substitute the 2-methylimidazole (MIM) ligand of ZIF-8, therefore enhancing the stability and catalytic activity while maintaining the crystal structure.

### MOF/carbon hybrid materials

The organic ligand in MOFs can be partially pyrolyzed during a thermal treatment process with improved absorption, catalytic or electrochemical performance [81–90]. Hence, the modification of carbon to MOFs may be considered one of the most important species for MOF hybrids.

For instance, infrared rapid thermal annealing technology was employed to treat Bi-MOF (CAU-17) subsequently generating the Bi_2_O_3_@C heterojunctions, where rich oxygen vacancies were introduced with negligible changes to the micromorphology to that of original CAU-17 [91]. It should be noted that the metal nodes can also participate in the pyrolysis and carbonization during the heat treatment, which is evident in the case of annealed bimetallic ZIFs [72]. Specifically, the micromorphology of Ni/ZIF-8 presented nanotube structure meanwhile that of Cu/ZIF-8 maintained its original shape, which was possibly ascribed to potential catalytic capacity of Fe, Co, Ni and other corresponding metals for the formation of carbon nanotubes [92–94].

Moreover, carbon dots can also be obtained from the phthalocyanine structure through a hydrothermal process, which is evident in the case of magnetofluorescent nanoparticles derived from Mn(II)-phthalocyanine as shown in Figure 2(E). Wang *et al*. [95] reported with regards to the self-assembly of Mn(II)-phthalocyanine-derived Mn-carbon dots with N-(Carbonyl-methoxypolyethylene glycol 2000)-1,2-distearoyl-sn-glycerol-3-phosphoethanolamine, sodium salt (DSPE-PEG) to form nanoparticles that could be useful for ameliorating tumor hypoxia and enhancing tumor photodynamic therapy (PDT) efficacy. Mn-CD was prepared by reacting the ethanol solution of Mn-MOF at high temperature (180°C) for 24 h. MOF-derived Mn-CD can catalyze *in situ* oxygen production from H_2_O_2_ and induce ^1^O_2_ production in response to light, which efficiently enhances PDT efficacy in hypoxic solid tumors, while acting as a fluorescence (FL)/magnetic resonance (MR) bimodal imaging agent to guide PDT (Figure 2(F–H)). Mn-CD has low biotoxicity and can be cleared from the body, making it a novel multifunctional nanomaterial for biomedical applications.

### Guest molecules

Metal nanoparticles such as metal oxides/sulfides/phosphorides, non-metal oxides, gas molecules, dyes and biomolecules (e.g. medicines, enzymes, amino acids), are among the guest molecules [96–101]. Guest molecules exist either as a cavity embedded in the MOF or encapsulated by the exterior of the MOF. The former, as the name implies, means that the guest occupies the pores of the MOF, while the latter means that the MOF gradually forms around the guest particles [4]. The assembly between MOFs and guest molecules not only retains the unique characteristics of MOFs but also decreases agglomeration of the guest particles, which is helpful for boosting the mechanical, electrochemical, adsorption and sensitivity qualities for a specific application [102–104].

**Figure 3.**
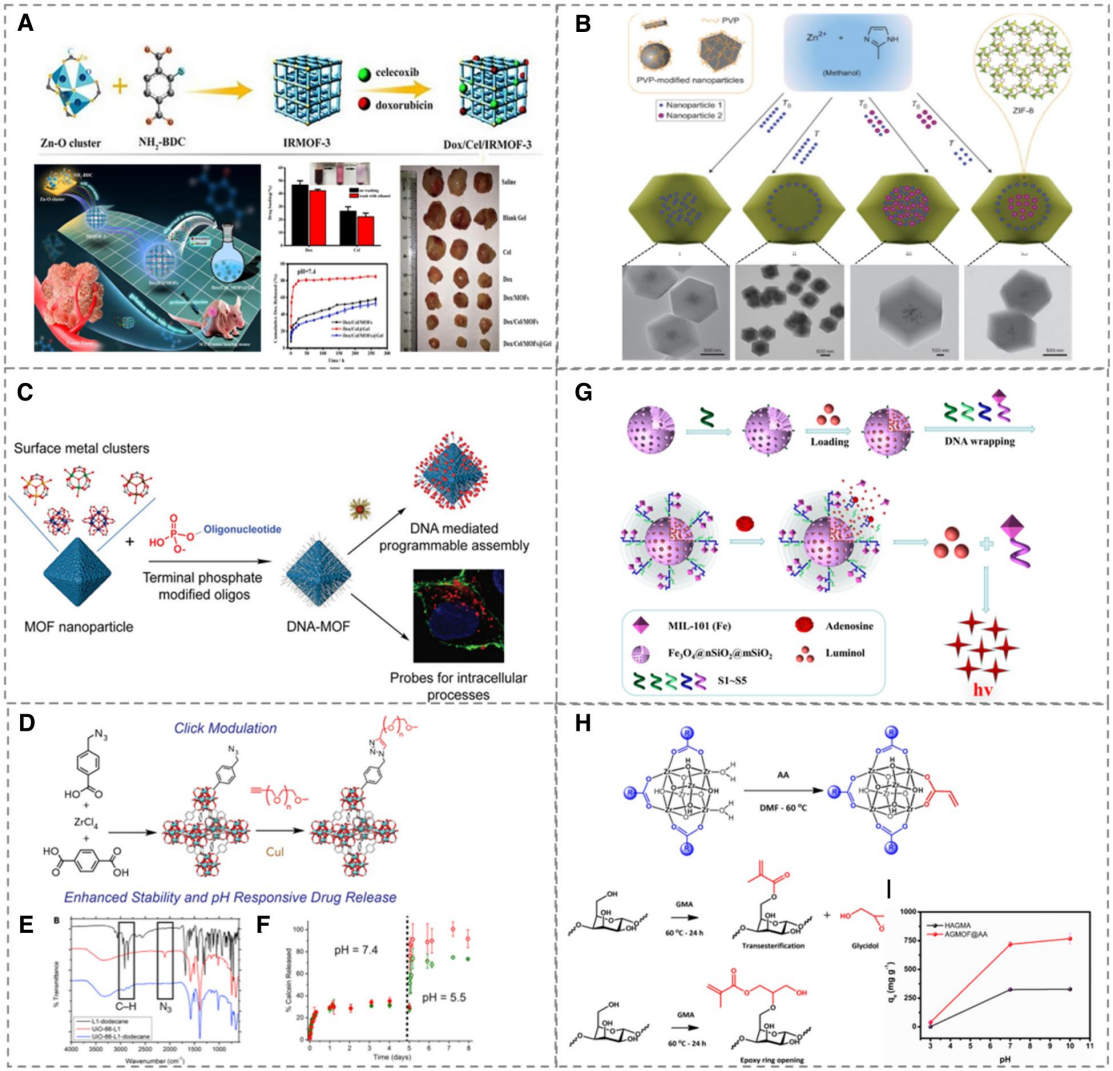
**(A)** Schematic overview of the preparation of dox/cel/MOFs@gel hydrogels with drug loading and slow release for tumor suppression. Reproduced from Ref. [106] with permission of Elsevier, ©2020. **(B)** Controlled encapsulation of nanoparticles in ZIF-8 crystals. Nanoparticles 1 and nanoparticles 2 are introduced at different time periods (reaction time T_0_, reaction period T). Reproduced from Ref. [114] with permission of Springer Nature, ©2012. **(C)** Functionalization of MOFs by oligonucleotides to promote cellular uptake of insulin. Reproduced from Ref. [127] with permission of American Chemical Society, ©2017. **(D)** Coupling reaction of alkyne on cross-linker molecules PEG with the azide groups of the modified MOF achieves response release of the drug. **(E)** Stacked IR spectrum (disappearance of –N_3_ and appearance of C-H on UiO-66-L1) and **(F)** pH-responsive release of calcein from the PEGylated MOFs. Reproduced from Ref. [135] with permission of Elsevier, ©2017. **(G)** Synthesis of DNA cross-linked hydrogel biosensor and schematic view of adenosine response. Reproduced from Ref. [151] with permission of Elsevier, ©2022. **(H)** Modification of MOFs by acrylic acid and gum Arabic by GMA. **(I)** Effect of pH on MB absorption profile of AGMOF@AA. Reproduced from Ref. [146] with permission of Elsevier, ©2019.

Direct immersion could be employed to functionalize MOFs with small guest molecules [96, 105], as demonstrated by Ren *et al*. [106]. Two popular small-molecule anticancer drugs, doxorubicin and celecoxib, were loaded onto IRMOF-3 utilizing the solvothermal synthesis process. As shown in Figure 3(A), the complex material is a promising vehicle for the local treatment of oral cancer when combined with a conventional injectable hydrogel in terms of pH-responsiveness, antitumor efficacy, biocompatibility, and the simultaneous release of hydrophobic and hydrophilic drugs at the oral tumor site.

In addition, it is feasible that the guest molecule may well be decorated into MOFs by loading with precursor through a particular reaction and under the appropriate conditions [104, 107–110]. Chen *et al*. bathed core–shell upconversion nanoparticle (UCNP)-iron porphyrinic MOF NPs in a solution of HAuCl_4_ which was then reduced by NaBH_4_ to produce UMOFs@Au NPs [111]. Notably, UMOFs@Au NPs have the catalytic activity of glucose oxidase-like (GOx) as well as avoid the instability and acute toxicity of GOx, which enhances the tumor starvation treatment. Similarly, Tang *et al*. [112] used KMnO_4_ to oxidize the –CH_3_ on Co-MOF and Zn-MOF ligands in order to produce MnO_2_/MOF-COOH with improved electrochemical characteristics.

Synthesizing MOFs by ‘as-prepared’ nanoparticles is another typical technique to generate hybrid materials. As a photosensitive NO donor, Bu *et al*. [113] coated pyrolytic UCNPs with ZIF-8 functionalized by S-nitrosothiols (CysNO). When irradiated by near-infrared light, UCNPs will emit ultraviolet light. The exciting ultraviolet light would break the S–NO link in CysNO and release NO out after, therefore providing on-demand gas therapy for the healing of spinal cord injuries. The spatial distribution of nanoparticles in the products within the MOFs varies when nanoparticles are inserted at distinct durations. Huo *et al*. [114] investigated the different spatial distributions of Au NPs within MOF crystals, as shown in Figure 3(B). When Au NPs were added at the early stage (T_0_) of synthesis, the MOF crystals could not yet be formed and the nanoparticles were more inclined to aggregate in the MOF cores. When the reaction was added for a period of time (T), the MOFs initially formed nuclei and the nanoparticles were more easily dispersed in the MOF transition layer. This construction with different spatial dispersion is suitable for applications where different particles are to be brought into the MOFs to achieve a temporal release of the guest particles.

### Polymers

Polymers have numerous desirable qualities including flexibility, stability, mechanical properties and the potential to react with existing MOFs. Some polymer chains may respond to external stimuli such as temperature or pH, which is crucial for on-demand therapy [115]. In addition, the dispersion and chemical stability of MOFs under physiological circumstances may be enhanced by polymer functionalization, allowing for many biological related applications [75, 116–118].

MOFs can be bound to polymers by non-covalent interactions such as hydrogen bonding and electrostatic mutual attraction [119–125]. Qiu *et al*. [126] completed the surface functional modification of Ni/Co-MOF to obtain pBDT@PDA-Ni/Co-MOF by a non-covalent modification strategy using dopamine and 1,4-benzenedithiol as polymer monomers. The modified MOF products had a hollow structure and were based on π–π electrostatic interactions to efficiently enhance the absorption of sulfonamides, providing a powerful tool for the detection of sulfonamides in water.

As illustrated in Figure 3(C), Mirkin *et al*. [127] employed nucleic acid-functionalized MOFs to facilitate insulin in crossing the cell membrane in order to increase protein internalization within cells. By ligating to the phosphate group on the oligonucleotide, the Zr-MOF central metal Zr^4+^ enables the oligonucleotide to wrap around the MOFs and establish a spatial barrier, so boosting the passage of MOFs@insulin into the cell. After co-culturing SKOV-3 cells with human ovarian cancer cells, laser confocal fluorescence imaging detected insulin enrichment in cell vesicles. This functionalized treatment enhanced insulin loading, enhanced the stability of MOFs@insulin in the physiological environment, and greatly enhanced insulin absorption by cells.

MOFs can also bind to polymers through covalent interactions [128, 129] (e.g. redox reactions). Wolfrum *et al*. [118] prepared three composites by utilizing post-synthetic polymerization to access dopamine, p-phenylenediamine and 3,4-ethylenedioxythiophene into Fe-MOFs cavities. In the reaction, Fe ions act as an oxidant as Fe^3+^ is reduced to Fe^2+^, oxidizing the amine/hydroxyl/thiophene ring in the corresponding monomer. The MOF polymerization product has high sensitivity in detecting H_2_O_2_ which can be used as an efficient peroxidase for biosensing applications.

In particular, click chemistry involving azide–alkyne, mercapto-alkyne and thiol-alkene has been used extensively to functionalize MOFs with organic molecules such as fluorescent dyes, modified DNA, peptides and polyethylene glycols [130–134]. Forgan *et al*. [135] modified p-azidomethylbenzoic acid with UiO-66 so that the azide could serve as a reactive site for bonding to alkynyl-terminated polyethylene glycol via click chemistry, thereby optimizing the dynamics of drug release and enhancing its stability in the physiological environment (Figure 3(D–F)).

### Hydrogel

Biocompatible MOF-hydrogel functionalized materials have excellent prospects for many applications in biosensing, adsorption and disease treatment [136–141]. Hydrogels commonly used for biological applications including polysaccharides, gelatin, silk protein, etc., have great value in combination with MOFs. They are biocompatible, biodegradable, enable delayed release of molecules and promote tissue repair [142–145]. Most biomolecules are thermosensitive or chemosensitive, thereby making simple and mild reaction conditions favorable for functionalization with MOFs.

There are a wide range of applications for hydrogels that are manufactured by the physical binding of MOFs and organic molecules [146–149]. Ding *et al*. [150] prepared amphiphilic composite hydrogels by hydrothermal reduction of reduced graphene oxide and MIL-101. The oxygen-containing functional groups on graphene were removed during hydrothermal reduction and then used as hydrophobic frameworks, while MIL-101 was used as a hydrophilic component. In addition, Wang *et al*. [151] constructed a highly sensitive and selective biosensor by incorporating nucleic acid-modified MIL-101(Fe) into a Fe_3_O_4_@SiO_2_-coupled polyacrylamide DNA chain cross-linked hydrogel (Figure 3(G)). In such biosensor, adenosine has been utilized to induce the production of the signaling molecule luminol by binding specifically to nucleic acids. Fe-MOF mimics the interaction of catalase with H_2_O_2_ to increase the chemiluminescence signal. As the adenosine concentration rises, the hydrogel shell is progressively dissolved allowing, the signal molecules enter the solution, and the luminescence signal becomes more intense. As a result, the composite hydrogel may be used for the detection of adenosine concentrations in various samples.

Rinaldi *et al*. [146] created composite hydrogels by covalently cross-linking MOFs with gum Arabic to generate innovative, highly absorbent frameworks for removing methylene blue (MB) from dye effluent. As depicted in Figure 3(H), UiO-66 is modified with acrylic acid through the aid of a solvent, while glycidyl methacrylate (GMA) alters the gel surface, followed by chemical polymerization to generate the cross-linked hydrogel AGMOF@AA. During MB adsorption studies, AGMOF@AA hydrogels exhibited pH-dependent adsorption rates ranging from 40.23 to 768.03 mg g^−1^ of MB at pH values between 3 and 10 (Figure 3(I)). Most carboxyl groups in the structure became ionized above pH 7.0, facilitating electrostatic adsorption between MB and the sample. Subsequent adsorption–resolution tests revealed that MB adsorbed under alkaline conditions and desorbed under acidic conditions, and that the hydrogel retained superior adsorption ability after five cycles.

## Multiple functionalization of MOFs in biomedical engineering

MOF-functionalized composites have interesting applications in the area of biomedical engineering [152–158]. The following sections review the applications of MOFs functionalization in tissue healing, illness therapy, bio-detection and imaging.

### Tissue restoration

Excessive inflammation, bacterial infection, impaired angiogenesis and cytokine/growth factor dysregulation impede the progression of several types of tissue healing [159–162]. The optimal medium for facilitating tissue healing and regeneration should be one which is biocompatible, able to control inflammation, stimulate angiogenesis and have a high level of bioactivity, thus promoting cell proliferation and differentiation. MOFs can significantly contribute to tissue healing processes by reducing inflammatory expression, stimulating angiogenesis, eliminating reactive oxygen species (ROS) and providing antibacterial abilities [163–169]. MOFs have largely been utilized for the repair and regeneration of bone, skin and nerves. The application of MOF-based materials in tissue repair is provided in Table 1, and some representative applications will be described.

**Table 1.** A summary of the MOF-based materials used in tissue restoration

MOF-based material	Specific role	Model	Ref.
GOx/CDs@MOF NFs	Monitor pH and anti-bacterial	Diabetic infection wound mice	[62]
UCNP@ZIF-8@SNO NPs	Load nitrosothiol and promote neuroregeneration	Clip-compression SCI rat	[113]
Mn-ZIF-8 NPs	Anti-bacterial and anti-inflammation, regulate immune cells polarization	Infection wound mice	[163]
PCL/Col/ZIF-8 membrane	pH-responsive release Zn^2+^, promote angiogenesis and osteogenesis	Calvarial defect rat	[164]
Au@ZIF-8/PVA-Alg hydrogel	PTT and POD-like activity, disrupt bacterial membrane and promote protein leakage	Diabetic infection wound rat	[165]
Mg-MOF-based MN-MOF-GO-Ag hydrogel	Scavenge ROS, anti-bacterial, promote angiogenesis	Diabetic wound mice	[166]
CCM@ZIF-8-PLLA scaffold	Anti-inflammation, anti-oxidant	Diabetic wound mice	[167]
Co-H4abtc particles	Activate PI3K/Akt signaling pathway	NSCs cell	[168]
Zn-BTC particles	Anti-bacterial, anti-inflammation and promote fibroblasts proliferation	Diabetic infection wound rat	[169]
IL4-Mg-MOF@CaP nanoplatform	Anti-inflammation, scavenge ROS, promote angiogenesis and osteogenesis	Calvarial defect rat	[170]
PLGA/Exo-Mg-GA MOF scaffold	Anti-inflammation, promote angiogenesis and osteogenesis	Calvarial defect rat	[171]
Mg/Zn-MOF74-titanium	Anti-bacterial, anti-inflammation and promote osteogenesis	Infection femurs rat	[177]
AHT-Ce/SrMOF implant	Scavenge ROS, restore mitochondrial functions, promote osteogenesis	OVX rat	[179]
MOFs@PVA hydrogel membrane	Anti-bacterial, anti-inflammation, promote angiogenesis and collagen deposition	Infection wound mice	[180]
FPS-ZM1@ZIF-67 NPs	Promote angiogenesis, anti-inflammation, attenuate high glucose-induced EC dysfunction	Diabetic wound rat	[181]
GelMA/HA-E/Ag@ MOF hydrogel	Anti-bacterial and anti-inflammation	Burn infection wound rat	[182]
QCS-OHA-(K-γ-CD-MOFs)-α-LA hydrogel	Anti-bacterial, self-healing, promote adhesion	Diabetic wound rat	[183]
QCS-Zn-MOF-Van hydrogel	Anti-bacterial, anti-inflammation, promote angiogenesis and nerve regeneration	Infection wound rat	[186]
Cu-TCPP-TCP scaffold	Promote angiogenesis and osteogenesis differentiation	Femoral defect rabbit	[197]

**Figure 4.**
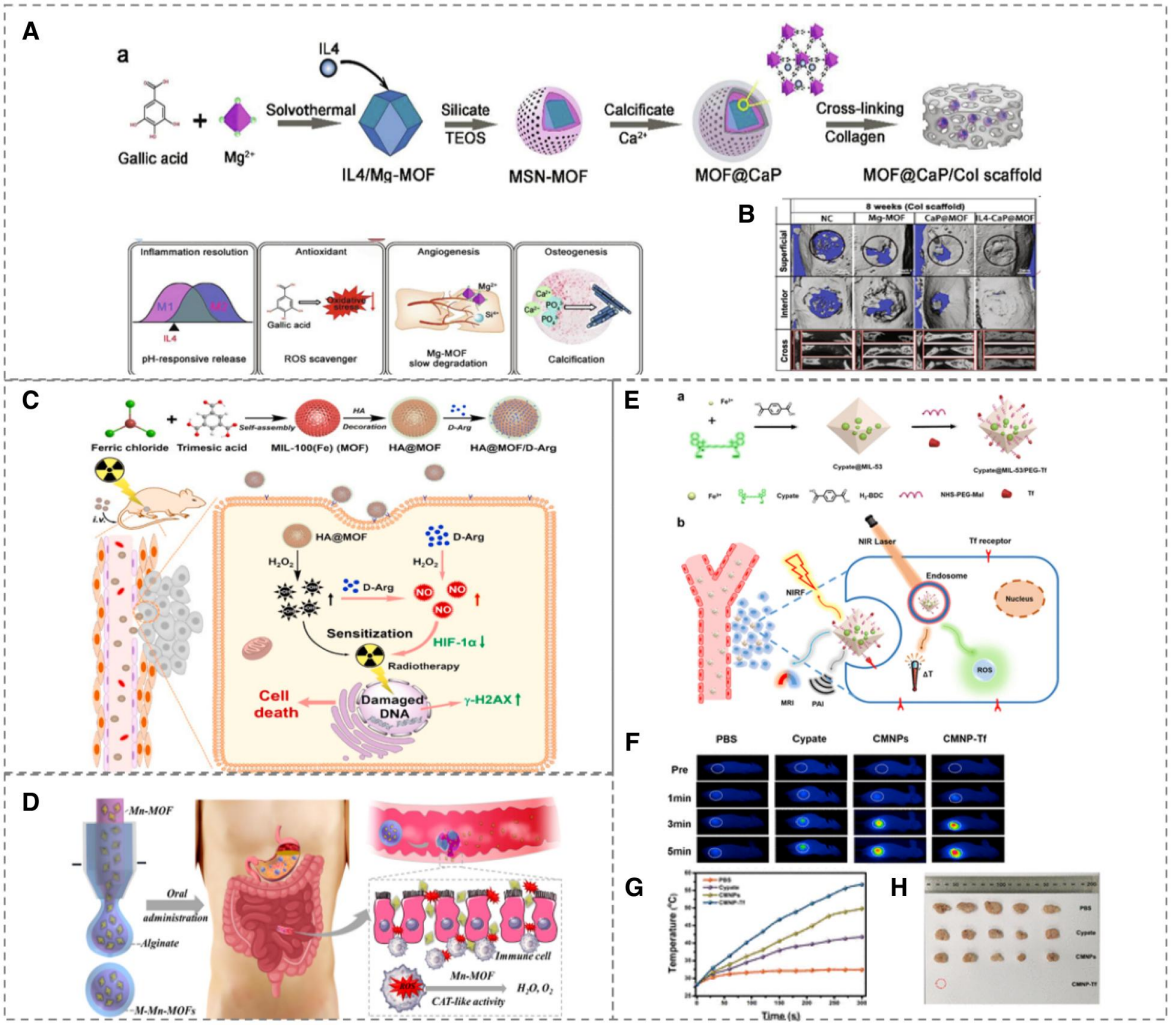
**(A)** Schematic diagram of L4-MOF@CaP scaffold formation and its exertion of immunomodulation, antioxidation, promotion of neovascularization and osteogenesis. **(B)** Micro-CT of rat cranial defects at 8 weeks after scaffolds implantation. Reproduced from Ref. [170] with permission of Elsevier, ©2020. **(C)** HA@MOF/D-Arg synthesis route and use for osteosarcoma radiotherapy. Reproduced from Ref. [196] with permission of Elsevier, ©2021. **(D)** Microfluidic synthesis of Mn-MOF microcapsules mimicking CAT for IBD treatment. Reproduced from Ref. [200] with permission of Elsevier, ©2022. **(E)** Schematic diagram of synthetic route of cypate@MIL-53/PEG-Tf and its use in the diagnosis and treatment of tumors, **(F)** infrared thermal images of CMNP-Tf under NIR laser irradiation, **(G)** corresponding tumor temperature elevation profiles and **(H)** final morphology of tumors after 14 days of treatment. Reproduced from Ref. [208] with permission of American Chemical Society, ©2019.

An important consideration in bone tissue healing is the ability of the substance to induce vascular regeneration so that the wounded region may receive adequate nutrients necessary for bone tissue regeneration in a timely and efficient manner. Metal nodes in MOFs may mediate angiogenic expression pathways required for efficient bone repair, taking Mg-MOF as an instance [170, 171]. MOF porosity makes it an attractive drug delivery vehicle and a good platform for combination pharmacological therapy to successful achieve angiogenesis. As seen in Figure 4(A), Wang *et al*. [170] employed Mg-MOF (Mg^2+^-gallate) as the MOF core, loaded with the anti-interleukin IL4 and wrapped with calcium phosphate to generate IL4-MOF@calcium phosphate nanospheres, which were then inserted into collagen to build a multifunctional biodegradable scaffold. The functionalized scaffold contains Mg^2+^ to promote angiogenesis, IL4 to mimic physiological inflammation regression and gallic acid to significantly eliminate excess ROS and attenuate ROS-induced inflammation in macrophages, thereby providing an optimal microenvironment for damaged bone repair. Furthermore, calcium phosphate shell may offer many osteogenic nucleation sites for multicellular bone regeneration. Similarly, Jiang *et al*. [171] generated a Mg-MOF to load exosomes obtained from human adipose-derived stem cells, which were then packed in a poly (lactic acid-co-glycolic acid) matrix to produce osteogenic scaffolds. Exosomes enhanced the adhesion, proliferation, osteogenesis and differentiation of human bone marrow mesenchymal stem cells and promoted the transfer of several angiogenic biomolecules, such as vascular endothelial growth factor.

In addition, alloy scaffolds have been utilized in clinical applications for bone substitutes, despite their vulnerability to corrosion, bacterial contamination and latent inflammatory response [172, 173]. MOF coating on the surface of alloys may provide antibacterial, anti-inflammatory and osteogenic activity while shielding the alloy [174–177]. For example, Li *et al*. [178] covered the surface of aminated magnesium alloy with a dense bio-MOF@Mg alloy coating consisting of Zn^2+^ with biphenyl dicarboxylic acid–adenine ligands; the stable and uniform MOF layer, as well as the mineralized hydroxyapatite layer, prevented the interaction between alloy and electrolytes in physiological conditions, thereby significantly extending the lifespan of bone implant scaffolds. Furthermore, Cai *et al*. [179] coated Ce/Sr bimetallic MOF onto Ti substrates through coordination effects, where Ce and Sr ions promoted osteogenic development of mesenchymal stem cells owing to lowered ROS levels and activating the AMPK signaling pathway.

The potential application of functionalized MOF/hydrogel composites in the field of wound repair is remarkable. Kong *et al*. [180] coupled Zn-MOF with polyvinyl alcohol (PVA) to develop a hydrophobic porous hydrogel wound dressing for infected skin wound healing which is resistant to blood, bodily fluids and bacterial contaminants. MOF embedded within hydrogel constantly delivered antimicrobial ions from the network to the site of infection in a controlled way, promoting wound healing through a synergistic interaction between antimicrobial and collagen deposition. Zheng *et al*. [181] loaded a receptor for advanced glycosylation end-product inhibitors (FPS-ZM1) into ZIF-67, where Co^2+^ activated HIF-1 to promote vascular regeneration while FPS-ZM1 attenuated vascular damage by mediating the transformation of macrophage phenotype, synergistically promoting chronic wound healing.

MOFs/hydrogel composites can also play an antimicrobial role in tissue repair. In order to reduce bacterial infection in burn wounds and accelerate wound healing, Chen *et al*. [182] devised GelMA/HA-E/Ag@MOF, a composite hydrogel system based on Ag@γ-cyclodextrin MOF, in which Ag@MOF acted as an antimicrobial agent to effectively reduce the bacterial population. The composite hydrogel serves as a potential material for accelerating burn wounds as it can accelerate epithelial regeneration and early angiogenesis while simultaneously regulate inflammation. Furthermore, Jiang *et al*. [27] designed an injectable hydrogel with antibacterial and anti-inflammatory properties for periodontitis by incorporating ZIF-8 nanoparticles loaded with the anti-inflammatory drug dexamethasone into a photocrosslinked hydrogel. ZIF-8 could release Zn^2+^ in response to an acidic environment and exhibits sustained antimicrobial activity. The hydrogel allowed injection into deep periodontal pockets of rats and presented excellent inhibitory properties against oral microflora, effectively reducing periodontal inflammation and mitigating inflammation-induced bone loss in rats with periodontitis and facilitating periodontal healing. In addition, Chen *et al*. [183] developed a chitosan and hyaluronic acid composite hydrogel scaffold doped with γ-cyclodextrin-MOFs loaded with α-lipoic acid (α-LA) for the repair of diabetic wounds in rats. The MOF delivered α-LA to the wound and sustained its release, maximizing the antimicrobial and antioxidant properties of α-LA. The composite hydrogel possessed excellent self-healing, adhesion and antibacterial properties, which promotes cell proliferation migration and collagen deposition, accelerating wound healing.

Additionally, numerous nerves lie under subcutaneous tissue, where nerve regeneration should also be taken into account during wound treatment [184, 185]. Dai *et al*. [186] encapsulated curcumin in Zn-MOF, which was subsequently incorporated into a quaternary ammonium salt chitosan hydrogel matrix, demonstrating synergism for promoting vascular and nerve regeneration via significant inhabitation for bacterial contamination, effective regulation of the macrophage phenotype and photothermal effect.

### Disease treatment

High specific surface area, adjustable porosity, tunable active sites and acceptable biocompatibility enable the MOF to integrate multiple therapies (such as drug therapy, PDT, photothermal therapy, chemodynamic therapy and gas therapy) for the treatment of disease [38, 44, 187–189]. In this section, an important applied direction of MOF-based materials lies in anti-tumor, and Table 2 presents their applications in disease treatment.

**Table 2.** A summary of the MOF-based materials used in disease treatment

MOF-based material	Specific role	Model	Ref.
MnTE-2-PyP^5+^ NPs	Anti-oxidant and anti-inflammation	AIA mice	[18]
ZIF-8-DEX@PPEMA-GelMA hydrogel	Anti-bacterial and anti- inflammation	Ligation-induced periodontitis rat	[27]
CaP@Fe-TCPP NPs	Immunotherapy	Breast cancer mice	[33]
HA-ZIF-8@DNAzyme NPs	Target tumor cells, decrease NAD^+^ and inactivate GAPDH, inhibit strong glycolysis, cut off glucose supply	B16-F10 tumor-bearing mice	[34]
FA-doped-MIL-100-UCNPs NPs	Oxidative stress and calcium overload	HeLa tumor-bearing mice	[36]
Fe-Cu-SS MOFs-Au NPs	RT and ferroptosis	Breast cancer mice	[37]
Mn-MOF-CDDP@PCM-TCM Nm	MW thermo-dynamic and chemotherapy	Lung cancer mice	[40]
BA@Zr-TCPP nano-co-crystals	Boron neutron capture therapy	Orthotopic glioma mice	[42]
Fe/Tm-MOFs/DOX particles	PTT, CDT and chemotherapy	Breast cancer mice	[45]
Gd-MOF-5 NPs	Immunotherapy	Breast cancer mice	[76]
Mn-CDs NPs	Ameliorate tumor hypoxia and PDT	Breast cancer mice	[95]
ZIF-8/DOX NPs	Load and sustained release of drugs	A549 lung carcinoma cell	[105]
Dox/Cel/IRMOFs-3@Gel hydrogel	Oral sustained drug delivery	Oral cancer rat	[106]
UiO-66-L1-PEG NPs	Enhance cell uptake, pH-responsive release drugs	HeLa cells	[135]
PEI-Mn(III)-TCPP@SAHA-AS1411 aptamer NPs	Damage and release nuclear DNA into cytoplasm, enhance innate immunity and synergize with PDT-induced ICD	Lung metastasis tumor mice	[187]
5-FU@α-CHC/AL/DCA @UiO-66 NPs	Drug delivery	MCF-7 cancer cells	[190]
Zr-MOF-P@Pt@P-Au-FA NPs	Ameliorate tumor hypoxia, PDT, starvation therapy	Breast cancer mice	[191]
Pd-MOF-H2 NPs	Hydrogen delivery and PTT	Breast cancer mice	[192]
Mg/HCOOH-MOF particles	Anti-inflammation, upregulate osteogenesis genes	MG63 cells	[193]
HA@MIL-100/D-Arg NPs	Deliver D-Arg, scavenge ROS, generated •OH	Osteosarcoma mice	[196]
Cu-TCPP-TCP scaffold	PTT	Bone tumor mice	[197]
Ex@MIL101@Gel NPs	Effective load exendin-4, intestinal pH-triggered self-unpacking	Diabetes rat	[198]
Mn_3_O_4_/UIO-TPP	Target mitochondria, restore ROS-induced damaged mitochondrial function	OA rat	[199]
Mn-MOF-encapsulated microcapsules	Relieve oxidative stress, anti-inflammation, restore intestinal barrier	IL-10-deficient mice (IBD)	[200]
Fe-MOF@Amp particles	Decompose H_2_O_2_, anti-bacterial	Pulmonary infection mice	[201]

Ren *et al*. [106] combined MOFs and temperature-sensitive hydrogels to develop an injectable composite material as a dual drug carrier for the treatment of oral tumors. The material exhibits high drug loading capacity, stable delivery, pH-responsive release and tumor suppression properties without significantly interfering with the physiological metabolic activity of healthy tissues. In addition, Forgan *et al*. [190] incorporated three drugs including carboxylates/phosphates into UiO-66 as modulators, which were dispersed at the defect sites by the MOF via coordination while preserving the original porosity features. A fourth drug was loaded into the pores of the MOF via adsorption, resulting in the release of multiple drug for use in anti-cancer treatment. Wu *et al*. [191] designed and synthesized a catalytic material composed of MOFs (Zr-porphyrin) and noble metal nanoparticles, where the Pt could catalyze the decomposition of hydrogen peroxide into O_2_ and singlet oxygen meanwhile singlet oxygen can be used to inhibit tumor regeneration and oxygen could promote Au NPs to decompose grapes. Through a cascade catalysis, the functional combination of photodynamic and hunger treatment was accomplished in this study, therefore preventing tumor renewal. He *et al*. [192] constructed a Pd-MOF nanoparticle with efficient hydrogen-carrying capacity and near-infrared photothermal effect for targeted slow-release delivery of hydrogen to the tumor region. Combining hydrogen therapy with thermal therapy enabled photoacoustic imaging-guided thermo-hydrogen therapy of mouse breast cancer, which has potential in precision cancer therapy applications.

In addition to serving as a medicinal carrier, metal ions in MOFs may modulate the physiological processes of cells in order to cure illness [193–195]. As illustrated in Figure 4(C), Hu *et al*. [196] produced a multifunctional nanomaterial, Fe-MOF loading d-arginine, which was then combined with hyaluronic acid hydrogel for osteosarcoma irradiation. MOFs not only functioned as nanocarriers for the transport of d-arginine to the tumor but they also released Fe^3+^ to form hydroxyl radical via the Fenton reaction, therefore dissolving and limiting tumor development. In addition, Wu *et al*. [197] developed a novel copper porphyrin scaffold covered with tricalcium phosphate under solvothermal conditions, demonstrating peri-injury revascularization, bone regeneration and efficient photothermal performance for bone tumor treatment under near-infrared light irradiation.

Functionalized MOFs may also be employed to treat osteoarthritis, diabetes, provide respiratory protection, and address disorders associated to ROS [193, 198, 199]. Figure 4(D) illustrates how that Zhao *et al*. [200] designed a carboxymethylcellulose-encapsulated Mn-MOF (Mn-phosphoserine) microcapsule for the treatment of inflammatory bowel disease using microfluidic technology. The capsule structure protected the stability of Mn-MOF in the tissue fluid, enabling Mn-MOF to reach the inflammatory bowel site smoothly and to be released slowly at the target site. In inflammatory intestinal regions, Mn-MOF catalyzed the reduction of excessive ROS by neutrophils and macrophages. Also, in order to combat a type of lung injury caused by bacterial infection, Wang *et al*. [201] constructed Fe-MOFs doped with ampicillin. The metal node Fe^3+^ of MOFs, catalyzed by the Fenton reaction of H_2_O_2_ secreted by Streptococcus pneumoniae, produced hydroxyl radical and ampicillin responsively released from the collapsed MOF framework was able to exert synergistic bactericidal effects through chemical kinetics and antibiotic therapy. By intravenous administration to mice with pneumonia, the microspheres were able to effectively accumulate in the lungs, reducing H_2_O_2_ and DNA damage, and prevent bacterial transmission, effectively protecting lung tissue.

### Biological detection and imaging

Bio-detection and bio-imaging technologies play a crucial part in clinical diagnosis, and functionalized MOFs do indeed have amazing potential in designing and improving sensitive detection of biomarkers due to their adjustable absorption kinetics and selective response to biomolecules [202–204]. Table 3 exhibits relevant studies of MOF-based materials for bioimaging and detection. Functionalized MOFs are currently used in a variety of laboratory tests for liver and kidney damage, tumor markers, cholesterol, biomolecules including insulin and glucose, and antibiotic allergies [205–209]. Wang *et al*. [210] utilized ZIF-8 loaded with gold nanoparticle-modified graphene in conjunction with DNAzyme decorated layered-branched hybridization chain reactions to achieve efficient electrochemical detection of interferon. Numerous nano-cavities in ZIF-8 provided additional immobilization sites for Au nanoclusters, thereby amplifying the electrochemical response. In addition, the luminescence sensing principle may be included into the MOF by the use of a specific metal-node design [210–212]. Yan *et al*. [213] developed and fabricated a MOF material comprising Eu and Tb ions with tunable luminescence color through engineered Tb^3+^–Eu^3+^ energy transfer, revealing a low detection limit and a broad detection range for l-cysteine in complicated physiological settings.

**Table 3.** A summary of the MOF-based materials used in biological detection and imaging

MOF-based material	Specific role	Model	Ref.
HRP@ZIF-8-EZ-READ transducer	Blood-based GBM diagnosis and subtyping	Human glioblastoma and GBM patient tumors	[39]
Mn-MOF-CDDP@PCM-TCM Nm	MRI	Lung cancer mice	[40]
TP-CHA-MOFs nanoprobe	Two-photon fluorescence imaging	HeLa and MCF-7 cells, tumor tissue/liver slices	[41]
BA@Zr-TCPP nano-co-crystals	FL and PET imaging	Orthotopic glioma mice	[42]
Fe/Tm-MOFs/DOX particles	T_2_-weighted MRI	Breast cancer mice	[45]
Mn-CDs NPs	FL and T_1_-weighted MRI	Breast cancer mice	[95]
DNA hydrogel-Fe_3_O_4_@ nSiO_2_@mSiO_2_-MIL-101-S5	Responsive release luminol and amplify biological signal	Detect adenosine in urine	[151]
Zr-MOF-P@Pt@P-Au-FA NPs	Track accumulation of NPs in tumors by near-infrared fluorescence imaging	Breast cancer mice	[191]
Pd-MOF-H_2_ NPs	Photoacoustic imaging and fluorescence imaging	Breast cancer mice	[192]
Fe-MIL-88B-NH_2_-NOTA-DMK6240/MB NPs	Target drug delivery, detect lesion area by MRI	Alzheimer’s disease rat	[207]
UiO-66-NDC NPs	Selectivity detect Fe^3+^	Fluorescent probe	[210]
AuNCs-GR@ZIF-8 NPs	Detect IFN-γion	Electrochemical biosensor	[211]
Eu@ZnMOF@SA fluorescent film	Simultaneously detect Asp and Arg	Fluorescent probe	[213]
Fe^3+^/Cu^2+^@Eu-K-MOF particles	Detect l-cysteine in serum	Luminescent probe	[214]
Mn-Ti MOFs@PEG nanosheets	MRI locate tumor and microwave treatment	HepG2 tumor-bearing mice	[218]
AuNRs@MIL-101-5-Fu@CP[5]A NPs	CT imaging and photothermal conversion	A2780 tumor-bearing mice	[219]

Bioimaging allows local visualization of lesions and thus accurate diagnosis and monitoring of disease appearance, progression and success of treatment. The application of MOFs in bioimaging well researched, where MOFs themselves can be involved in a variety of imaging for diagnosing and treating diseases or can be used as carriers of fluorescent probes for effective delivery to lesion sites [214–217]. MOFs are emerging in the disciplines of optical imaging, magnetic resonance imaging (MRI), computed tomography (CT) and other biological imaging, such as Mn-MOF, Fe-MOF and fluorescent MOFs [204, 218]. Yang *et al*. [208] loaded MIL-53 with a near-infrared dye that combines tumor tissue targeting with polyethylene glycol and transferrin (Figure 4(E–H)), allowing the integration of multimodal imaging (near-infrared fluorescence imaging, photoacoustic imaging, MRI) and photothermal/photodynamic therapy for the treatment of tumors. Similarly, Yang *et al*. [219] encased gold nanorods in MIL-101 coupled with supramolecular gates to allow CT imaging and stimuli-responsive drug release.

In order to monitor miRNAs which can provide critical information for the production and progression of various cancers, Lin *et al*. [41] developed a novel fluorescent nanoprobe (TP-CHA-MOFs) for amplified miRNA sensing that utilized UiO-66 as a DNA carrier with hairpins H1 and H2-TP-BHQ1 modified on the surface as two-photon TP fluorescent signal amplifiers. After the probe was internalized by the cell, the metal node Zr can be coordinated with intracellular phosphate to release H1 and H2-TP-BHQ1. Subsequently, the intracellular miRNA triggers the formation of H1–H2 double-stranded bodies, which turns on the two-photon fluorescence signal for the amplification detection of target miRNAs. This nanoprobe had sufficient cellular permeability and achieved high imaging performance of endogenous miRNA in deep tumor tissues, which has a high prospect of application in medical diagnosis.

In addition, research on the integration of MOFs for tumor detection and treatment is also gaining attention. Yu *et al*. [40] reported a specifically targeted multifunctional drug delivery system based on Mn-MOFs. Mn-MOFs have MRI and microwave thermal sensitization functions. The thiol groups on its ligands are oxidized and linked to form disulfide bonds, which can react with glutathione within the tumor. The composite microspheres are enriched in the tumor area under targeted action, and are locally heated by microwave irradiation to scavenge glutathione and degrade cisplatin for release. The degraded Mn^2+^ could catalyze H_2_O_2_ to produce •OH to enhance the anti-tumor effect. The product has a high T1 imaging capability for use as an MRI agent to provide tumor diagnosis and monitor treatment.

The great tunability and multifunctionality bring great potential for the application of MOF-functionalized materials in the field of biomedical engineering. In tissue restoration, an essential application of MOFs lies in its use as a drug delivery carrier to achieve efficient loading and controlled release of drugs by adjusting the pore size and surface properties, and its pH sensitivity also contributes to the release response of drugs. In addition, the porous structure of MOFs facilitates cell adhesion, proliferation and differentiation, and establishes a microenvironment that is conducive to tissue regeneration. MOF also has immunomodulatory properties and can promote tissue healing by modulating macrophage polarisation. Coordination metal ions of MOFs, including Zn^2+^, Mg^2+^ and Cu^2+^, can also play an active role in tissue repair, such as antibacterial, and promote vascular and nerve regeneration. In disease treatment, MOFs can be rationally designed to achieve precise treatment and repair of specific diseases. As a kind of good drug carrier, targeting function can be achieved by surface modification of MOFs to enhance drugs enrichment in diseased tissues. MOF-functionalized materials can also act as bionic enzymes to remove excessive local ROS, thus improving the pathological microenvironment, such as Cu-MOF, Ce-MOF, Fe-MOF, Mn-MOF and many other MOFs. MOF-functionalized materials’ high porosity and excellent adsorption capacity can provide more active sites for signal recognition molecules and signal enhancement molecules. The metal nodes and ligand functional groups of MOFs may identify the target and are used to support the selectivity and stability of the detection. MOFs with intrinsic luminescent properties, including rare-earth metal-MOFs or MOFs utilizing porous structures to accommodate fluorescent dyes, have found applications in fluorescence imaging. MOFs, such as Mn-MOF and Fe-MOF, are paramagnetic and ferromagnetic, have the potential to be used as contrast agents. By modulating metal ions and organic ligands, the stability and sensitivity of the material’s imaging signal can be enhanced. On this basis, the possibility of obtaining more imaging functions on the basis of the original imaging functions can also be realized by compounding other materials in the MOFs.

## Summary and prospects

Various functionalized modifications of MOFs with guest molecules, polymers, hydrogels, etc., have boosted the practical utilization MOFs. The incorporation of functionalized MOF materials in several fields, such as catalysis, adsorption, energy storage and biomedicine, will be investigated in more detail in future studies. In the biomedical field, the construction of MOF-functionalized materials has combined organic molecules, inorganic molecules and biomolecules to greatly exploit their respective advantages, resulting in significant research advancements in single or multiple research directions, including tissue repair, cancer therapy, detection and imaging.

The breadth and depth of investigations on the modalities of MOF functionalization have not yet been sufficient, and the primary obstacles for future advancement are outlined below.

### Controllable synthesis for MOF-based heterostructure

The enormous lattice mismatch and unpredictable nucleation and growth rate of MOFs make programmed design challenging. Future studies must therefore concentrate on the nucleation and growth process of MOF on the surface of functional nanomaterials, combining advanced *in situ* characterization techniques with theoretical calculations, such as molecular dynamics and density functional theory.

In addition, it is crucial to emphasize that the coordination center may interfere with free radical polymerization, hence inhibiting the *in situ* composite of MOF and polymer materials. The coating of biodegradable materials, such as bioactive glass, which can degrade in the physiological environment to produce ions to promote tissue repair and expose the coordination center, thereby realizing the effective combination of ion therapy and MOF materials, is one potential solution to this problem. The integration of MOFs with bioactive glasses is beneficial for achieving advantageous multiplier effects but has not yet been reported in studies. It is expected that the combination of MOFs and bioactive glasses will bring new breakthroughs in biomedical materials, which will be of great significance for the advancement of human biomedicine.

### Diagnosis and treatment of deep lesions

In the future, the NIR II window will play a greater role in the identification and therapy of deep lesions. The targeting ability of anti-tumor materials is an important factor in the therapeutic efficacy, and functionalization is a substantial method for enhancing MOF’s tumor targeting capacity. Future material design must take into account various targeting techniques.

### Enzyme-like catalysis

As stated before, the catalytic activity of MOFs has several biological uses, including but not limited to tumor therapy and tissue healing. Due to the fact that the pH, temperature and metabolic activity of cells are vastly different from laboratory settings, it is necessary to monitor the catalytic reaction pathway in a holistic physiological environment using advanced *in situ* dynamic characterization systems.

### Biocompatibility

Biocompatibility is a necessary precondition for the clinical translation of bio-materials. Although MOF materials have shown minimal toxicity or side effects in short-term cell/animal models *in vitro* in previous publications, a small number of MOFs or too high a concentration of MOFs may pose some potential toxicity problems. The ligand metals of mof, such as Cu, Mn, Co, Zn, Fe, Al, Cr, Zr and Mg, have different influences on its overall toxicity. Among them, the toxicity of MOFs coordinated by Cr, Zr and Mg is low, while the toxicity of MOFs coordinated by Cu and Mn is relatively high. And part of the reason for the cytotoxicity brought by MOFs is oxidative stress. Excessive ROS may activate inflammatory signaling leading to mitochondrial membrane, protein and mitochondrial DNA damage, which in turn leads to cellular damage and apoptosis. In addition, MOFs in antimicrobial applications may disrupt the integrity of cell membranes while disrupting bacterial membranes to inhibit bacterial growth, leading to alterations in membrane permeability and cellular homeostasis. The particle size of MOFs is also one of the factors to be considered for biomedical applications. Nano-MOFs lead to better positive results. It may be due to the fact that nano-MOFs have higher surface area and reactive sites for better drug loading, drug delivery and controlled release. If the particle size is too small, it may cross the blood barrier and enter the bloodstream. The smaller the particle size, the more toxic it may be. Their long-term accumulation and clearance in various animal organs cannot be ignored. Therefore, the immunogenicity, biocompatibility and clinical toxicity of the MOF materials must be rigorously evaluated over the long term.
